# CDK5RAP3 Is a Novel Repressor of *p14^ARF^* in Hepatocellular Carcinoma Cells

**DOI:** 10.1371/journal.pone.0042210

**Published:** 2012-07-31

**Authors:** Grace Wing-Yan Mak, Wai-Lung Lai, Yuan Zhou, Mingtao Li, Irene Oi-Lin Ng, Yick-Pang Ching

**Affiliations:** 1 Department of Anatomy, Li Ka Shing Faculty of Medicine, The University of Hong Kong, Hong Kong, China; 2 Department of Pathology, Li Ka Shing Faculty of Medicine, The University of Hong Kong, Hong Kong, China; 3 State Key Laboratory for Liver Research, The University of Hong Kong, Hong Kong, China; 4 Department of Pharmacology and the Proteomics Center, Zhongshan School of Medicine, Sun Yat-sen University, Guangzhou, Guangdong, China; The University of Hong Kong, China

## Abstract

CDK5 regulatory subunit associated protein 3 (CDK5RAP3) is a novel activator of PAK4 and processes important pro-metastatic function in hepatocarcinogenesis. However, it remains unclear if there are other mechanisms by which CDK5RAP3 promotes HCC metastasis. Here, we showed that in CDK5RAP3 stable knockdown SMMC-7721 HCC cells, p14^ARF^ tumor suppressor was upregulated at protein and mRNA levels, and ectopic expression of CDK5RAP3 was found to repress the transcription of *p14^ARF^*. Using chromatin immunoprecipitation assay, we demonstrated that CDK5RAP3 bound to *p14^ARF^* promoter *in vivo*. Furthermore, knockdown of p14^ARF^ in CDK5RAP3 stable knockdown HCC cells reversed the suppression of HCC cell invasiveness mediated by knockdown of CDK5RAP3. Taken together, our findings provide the new evidence that overexpression of CDK5RAP3 promotes HCC metastasis via downregulation of *p14^ARF^*.

## Introduction

CDK5RAP3 (also called C53/LZAP) was first identified as a binding partner of cyclin-dependent kinase 5 (CDK5) activator, p35^nck5a^, in yeast two-hybrid screening [1]. Several studies have investigated the potential roles of CDK5RAP3 in carcinogenesis, but so far its definite roles remain controversial. CDK5RAP3 has been found to promote apoptosis induced by genotoxic stress in HeLa cells by triggering G_2_/M arrest [2]. In addition, CDK5RAP3 has been proposed to be a tumor suppressor because it inhibits the NF-κB cell survival pathway and its protein level is significantly underexpressed in head and neck squamous cell carcinomas [3]. CDK5RAP3 can interact with a well-known tumor suppressor, namely alternate reading frame (p14^ARF^), by which stabilizes and promotes the transcription activity of p53 [4]; yet, the role of this interaction in carcinogenesis has not been explored.

More recently, we demonstrated that *CDK5RAP3* is frequently overexpressed in human HCCs and contributes to HCC metastasis by activating PAK4 [5]. CDK5RAP3 has 2 putative LXXLL motifs, which are the signature motifs for transcriptional co-regulators, mediating the binding on nuclear receptors. In addition, CDK5RAP3 has a leucine zipper domain, which is a structural motif for protein dimerisation and is commonly found in proteins involving in gene expression. Previous study has shown that CDK5RAP3 can associate with a nuclear co-activator, cAMP response element-binding protein (CREB)-binding protein (CBP) [6], suggesting that CDK5RAP3 may also function as a transcriptional co-activator/repressor. Here, we provide evidences that CDK5RAP3 is a putative transcriptional suppressor of *p14^ARF^* and overexpression of CDK5RAP3 promotes to the metastasis of HCC by downregulating *p14^ARF^*. Thus inhibition of CDK5RAP3 can potentially be used to restore the expression of the important tumor suppressor p14^ARF^ expression, providing new molecular targets for the therapeutic intervention in HCC and possibly other cancers.

## Materials and Methods

### Antibodies

Anti-CDK5RAP3 antibody was reported previously [5]. Rabbit anti-GFP (FL), mouse anti-Myc (9E10), rabbit anti-Myc (A-14), mouse anti-p53 (DO-1), mouse anti-phospho-p53 (S15) and rabbit anti-MDM2 (C-18) were purchased from Santa Cruz Biotechnology. Rabbit anti-p14^ARF^ was purchased from Abcam. Mouse anti-β-actin (AC15) was purchases from Sigma-Aldrich.

### Plasmids

The *p14^ARF^* promoter luciferase reporter construct, pGL3-p14^ARF^-luc, and the truncation mutants generated from pGL3-p14^ARF^-luc were generous gifts from Dr. Kiyoshi Ohtani [7]. Plasmid expressing Myc-CDK5RAP3 was reported previously [5] and pEGFP-p14^ARF^ was constructed by subcloning full length of p14^ARF^ cDNA fragment (clone IMAGE: 6173590) into pEGFP vector (Clontech, Palo Alto, CA). HA-E2F1 expression plasmid was obtained from Addgene (Sellers *et al.,* 1998, Addgene plasmid 10736).

### Cell Culture

Human hepatoma cell line HepG2 and monkey kidney fibroblast cell line COS7 were purchased from ATCC. Human HCC cell line SMMC-7721 was gift from Shanghai Institute of Biochemistry and Cell Biology, Chinese Academy of Sciences [8]. Cells were maintained in DMEM high glucose (Life Technologies, Rockville, MD) supplemented with 1 mM sodium pyruvate and 10% heat-inactivated FBS (JRH Biosciences, Lenexa, KS). Cells were transfected with DNA constructs using LipofectAMINE 2000 (Invitrogen, Carlsbad, CA) according to the manufacturer’s protocol.

### siRNA Oligonucleotide Transfection

CDK5RAP3 small interfering RNA (siRNA) duplex was transfected into cells as described [5]. siRNA duplex targeting *p14^ARF^* 5′-GCGGAAGGUCCCUCAGACAUU-3′ (sense strand) and non-targeting siRNA duplex negative control, 5′-UAAGGCUAUGAAGAGAUAC-3′ (sense strand) were purchased from Dharmacon, Inc, Lafayette, CO.

### Reverse Transcription-PCR (RT-PCR)

Quantitative real-time PCR (qPCR) was performed as described [9]. The sequence of taqman probes (Applied Biosystems) for *CDK5RAP3* and *p14^ARF^* are 5′-AGGAAAGATGGAGGACCATCAGCAC-3′ and 5′-TAGAAGACCAGGTCATGATGATGGG-3′ respectively; cellular 18S rRNA was used as an internal control.

**Figure 1:**
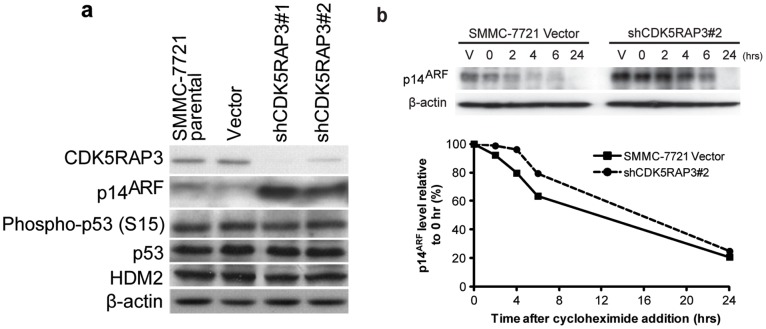
Regulation of p14^ARF^ localization and protein expression by CDK5RAP3. (**a**) The p14^ARF^ and CDK5RAP3 protein levels in stable CDK5RAP3 knockdown SMMC-7721 clones (shCDK5RAP3#1 and #2), vector control and parental cells [5] were compared by Western blotting using indicated antibodies, respectively. (**b**) The stable CDK5RAP3 knockdown SMMC-7721 (shCDK5RAP3#2) and vector control cells were treated with 100 µg/ml cycloheximide or DMSO (vehicle), and harvested at the indicated time points. p14^ARF^ and *β*-actin protein levels were determined by Western blotting. V: DMSO treatment. *Top*: Western blotting; *bottom*: quantification of p14^ARF^ protein level.

### Cell Migration and Invasion Assay

Transwell assay and invasion assay were performed as described [10], [11], respectively. The cells were allowed to migrate for 16 hours and invade for 24 hours, respectively. A total of 5 fields were counted for each filter. The experiments were performed three times independently.

### Confocal Microscopy

Cells were fixed with 4% paraformaldehyde and permeabilized with 0.2% Triton-X100 [9]. Images were captured by Carl Zeiss LSM510 (HKU Faculty core facility).

### Luciferase Reporter Assay

Cells cultured in 24-well plates were transfected with Myc-CDK5RAP3 in addition to 100 ng of p14^ARF^-luc or p53-luc, and 10 ng of pRL-CMV. Luciferase activity was measured using the Dual-Luciferase® Reporter Assay System (Promega, Madison, WI), and light emission was quantified using a microplate luminometer (MicroLumat PLUS, Perkin-Elmer, Shelton, CT).

### Chromatin Immunoprecipitation Assay

Chromatin immunoprecipitation (ChIP) assays were performed using a ChIP Assay Kit (Millipore, Billerica, MA). CDK5RAP3 stable overexpression clone #2 HepG2 cells (1×10^7^) were used for the assay.

### Statistical Analysis

Student’s *t*-test was used for statistical analysis of data. Tests were considered significant with *P*<0.05.

## Results

### Knockdown of CDK5RAP3 Upregulated p14^ARF^

Our previous study demonstrated that overexpression of CDK5RAP3 promoted HCC metastasis by activation of PAK4 [5]. However, as CDK5RAP3 is associated with tumor suppressor, p14^ARF^ [4], we wonder if *p14^ARF^* also plays a role in CDK5RAP3-mediated HCC formation. To investigate if CDK5RAP3 regulates p14^ARF^, Western blotting was performed to detect the protein level of p14^ARF^ in CDK5RAP3 stable knockdown SMMC-7721 cell lines [5]. Interestingly, the stable knockdown of CDK5RAP3 in SMMC-7721 cells resulted in an upregulation of p14^ARF^ (Fig. 1a). However, no significant difference was observed for the other components of the p14^ARF^/HDM2/p53 pathway, such as HDM2 and p53, suggesting that the upregulation of p14^ARF^ may be independent of the protein interaction between HDM2 and p14^ARF^ (Fig. 1a). Since the protein level of p14^ARF^ is largely regulated by protein degradation [12], the possibility that CDK5RAP3 might enhance the turnover rate of p14^ARF^ was examined. By treatment with cycloheximide, an inhibitor of protein synthesis, we observed that the protein degradation rate of p14^ARF^ in the CDK5RAP3 stable knockdown clone #2 was slower than that of the vector control cells in the first six hours of treatment, but they reached to a similar rate after 24 hours, suggesting that CDK5RAP3 only has a very transient effect on the protein stability of p14^ARF^ (Fig. 1b).

### CDK5RAP3 Repressed p14^ARF^ Transcription in HCC Cells

**Figure 2:**
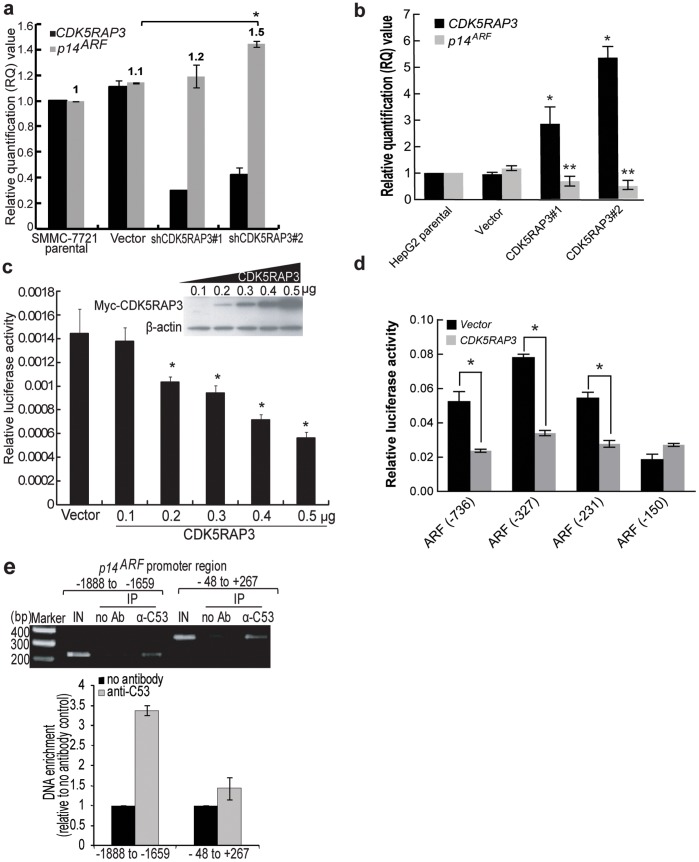
Suppression of endogenous expression of p14^ARF^ by CDK5RAP3. (**a**) The *p14^ARF^* and *CDK5RAP3* mRNA expression in stable CDK5RAP3 knockdown SMMC-7721 stable clones was determined by Quantitative real-time PCR (qPCR). Data was analyzed by comparative Ct method. Band intensity was analyzed using AlphaEasePC software and normalized with *β-actin*. Results were mean of three independent experiments. *, *P*<0.005, Student’s *t*-test. (b) Similar to (a), the CDK5RAP3 stable expressing HepG2 clones (CDK5RAP3#1 and #2), vector control and parental cells were used for qPCR assay. Results were mean of three independent experiments. *P<0.04 and **P<0.02 compared with vector control, Student’s *t*-test. (c) The CDK5RAP3 expression construct and p14^ARF^ luciferase reporter, pGL3-p14^ARF^ were co-transfected into SMMC-7721 cells for dual-luciferase reporter assay. Results represent mean ±SD for triplicate wells. *, *P*<0.05 compared with vector control, Student’s *t*-test. (d) Similar to (c), luciferase reporters carrying truncation mutants of the p14^ARF^ promoter, CDK5RAP3 expression construct (0.3 µg) and vector (0.3 µg) were used for dual-luciferase reporter assay. Results represent mean ±SD for triplicate wells. *, *P*<0.02 compared with vector control, Student’s *t*-test. (e) CDK5RAP3 bound *p14^ARF^* promoter by performing chromatin immunoprecipitation (ChIP) analysis on CDK5RAP3 stable overexpression clone #2 HepG2 cells (1×10^7^). Input (IN) and no antibody control (No Ab) were included. Error bars: mean ±SD.

**Figure 3:**
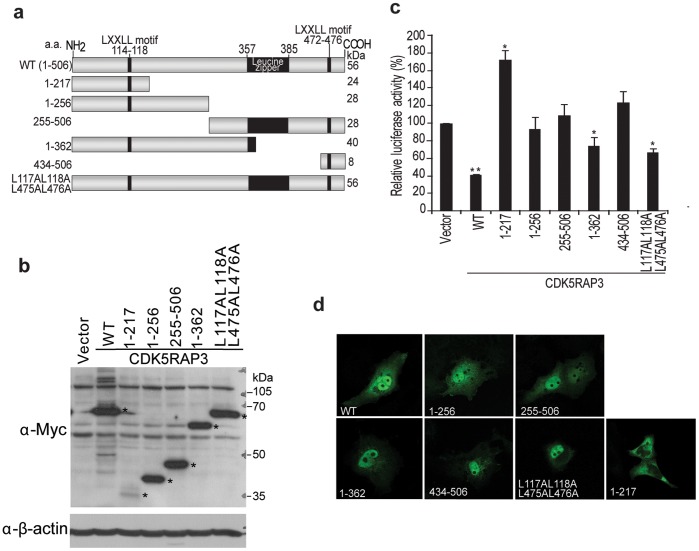
Nuclear localization of CDK5RAP3 was important for the suppression of p14ARF promoter activity. (**a**) *S*chematic diagram of CDK5RAP3 mutants (**b**) Western blotting showing the expression levels of CDK5RAP3 mutants overexpressed in HepG2. Protein lysates from reporter assay were used for Western blotting probed with anti-Myc antibody. (**c**) *D*ual luciferase reporter was performed by co-transfection of CDK5RAP3 mutants with p14^ARF^-luc reporter in HepG2. Results were mean of three independent experiments, with promoter activity of vector control set as 100%. *, *P*<0.05 and **, *P*<0.005 compared with vector control, Student’s *t*-test. (**d**) Confocal images of wild type (WT) and the indicated deletion mutants of Myc-CDK5RAP3.

To further understand how CDK5RAP3 might enhance the expression of p14^ARF^, and as CDK5RAP3 contains structural motifs suggested to be a transcriptional regulator, we examined if CDK5RAP3 regulates p14^ARF^ transcriptionally. *p14^ARF^* mRNA level was found to be upregulated in the CDK5RAP3 stable knockdown SMMC-7721 clones (Fig. 2a). Consistently, downregulation of *p14^ARF^* mRNA expression was also observed in two CDK5RAP3 stable overexpressing HepG2 clones that we established previously [5] (Fig. 2b). To further understand the mechanism by which CDK5RAP3 regulates *p14^ARF^* transcription, luciferase reporter assay was performed to determine the effect of CDK5RAP3 on *p14^ARF^* promoter transcription activity. A p14^ARF^ promoter luciferase reporter plasmid contained the *p14^ARF^* promoter region 736 base pair (bp) upstream from the transcription start site was used for the assay [7]. As shown in Fig. 2c, ectopic expression of CDK5RAP3 significantly suppressed the *p14^ARF^* promoter luciferase activity in a dose-dependent manner in SMMC-7721 HCC cells (Fig. 2c). Similar result was also observed in HepG2 cell line, suggesting that CDK5RAP3 can transcriptionally repress the expression of p14^ARF^ (Fig. 3c). To further understand how CDK5RAP3 suppresses p14^ARF^ transcription, we used luciferase reporters carrying a series of truncation mutants of the p14^ARF^ promoter to map the region responsible for the CDK5RAP3 suppression. As shown in Fig. 2d, CDK5RAP3 suppressed the luciferase activity of the p14^ARF^ promoter in the constructs of −736, −327 and −231 to roughly 50%, but not in the construct of −150, suggesting that nucleotides −231 to −151 are important for CDK5RAP3 suppression.

### CDK5RAP3 Bound to Endogenous p14^ARF^ promoter

As the ability of CDK5RAP3 to attenuate the *p14* ^ARF^ promoter activation and to repress endogenous *p14* ^ARF^ expression were demonstrated, we further investigates whether CDK5RAP3 binds to endogenous *p14* ^ARF^ promoter by chromatin immunoprecipitation (ChIP) assay using CDK5RAP3 stable overexpressing HepG2 cells [5]. Two sets of primers that has been reported previously were used to amplify *p14* ^ARF^ promoter regions (−1888 to −1659 and −48 to +267) [13]. As shown in Fig. 2d, immunoprecipitated with anti-CDK5RAP3 antibody significantly enriched the DNA fragments containing the promoter region of *p14* ^ARF^, as compared with the no antibody control (Fig. 2e), strongly indicating that CDK5RAP3 can direct bind to the *p14^ARF^* promoter.

### Nuclear Localization of CDK5RAP3 was Important for the p14^ARF^ Transcriptional Repressive Activity

To map out whether specific region of CDK5RAP3 is required for the repression of *p14^ARF^* transcription, a panel of deletion and LXXLL point mutation mutants of CDK5RAP3 was generated. To rule out the possibility that these mutants are unstable within the cells, Western blot analysis was performed to confirm the expression of these mutants. The result showed that all the mutants expressed at similar levels in cells, apart from the 1–217 deletion mutant, which expressed at a lower level (Fig. 3b). To examine the transcriptional suppressive activity of these mutants, luciferase reporter assay was performed. Mutation of both LXXLL motifs on CDK5RAP3 to LXXAA (L117AL118A/L475AL476A) and a 1–362 deletion mutant of CDK5RAP3 was much less potent in repressing the *p14^ARF^* promoter activity (Fig. 3c), as compared to the full length protein. Similar result of double point mutant was also obtained for single LXXLL/LXXAA mutants (data not shown). However, for the deletion mutants, including 1–256, 255–506 and 434–506, all of them had completely lost their repressive activity on *p14^ARF^* promoter (Fig. 3c). This result suggests that the overall integrity of CDK5RAP3 protein may be important for the repression activity. Surprisingly, the 1–217 mutant, which was relatively unstable, did not repress, but activates the *p14^ARF^* promoter activity (Fig. 3c). More interestingly, among the panel of mutants, the a.a. 1–217 mutant was the only mutant that did not localize to the nucleus (Fig. 3d), indicating that the nuclear localization of CDK5RAP3 may be important for the its repressive activity on *p14^ARF^* promoter and protein stability (Fig. 3d). Taken together, our data indicate that CDK5RAP3 repressed *p14^ARF^* promoter activity in HCC cells.

### CDK5RAP3 had no Effect on E2F1-mediated p14^ARF^ Promoter Transactivation

**Figure 4:**
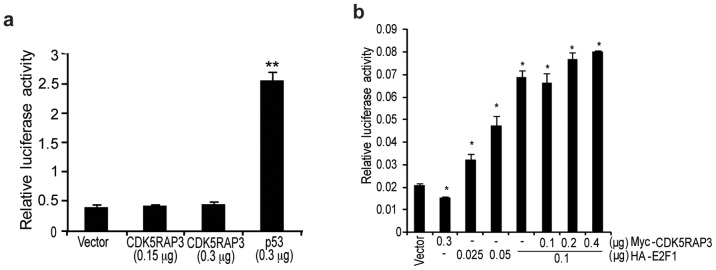
CDK5RAP3 transcriptionally regulated p14^ARF^ in a E2F1 independent manner. (**a**) CDK5RAP3 was co-transfected with p53-responsive element reporter for luciferase assay in HepG2 cells. Results represent mean ±SD for triplicate wells. *, *P*<0.05, **, *P*<0.005 compared with vector control, Student’s *t-*test. (**b**) Expression constructs of CDK5RAP3 was co-transfected with HA-E2F1 and p14^ARF^-luc reporter for luciferase assay in HepG2 cells. Results represent mean ±SD for triplicate wells. *, *P*<0.005 compared with vector control, Student’s *t*-test.

p14^ARF^ is the upstream activator for p53 activity as it can abrogate the MDM2-mediated degradation of p53 by inhibiting MDM2 [14], [15], we wonder if regulation of *p14^ARF^* expression by CDK5RAP3 affects p53 transactivation activity. Using p53 luciferase reporter assay, we showed that CDK5RAP3 did not affect the p53 promoter activity (Fig. 4a), indicating that CDK5RAP3 may not have an effect on p14^ARF^-mediated regulation of p53 in HCC cells. To examine if CDK5RAP3 represses the *p14^ARF^* via regulating the transcriptional activator of *p14^ARF^*, E2F1, luciferase reporter assays were performed by overexpressing Myc-tagged CDK5RAP3 and HA-tagged E2F1 in HepG2 cells. Although ectopic expression of E2F1 caused a dose-dependent activation of p14^ARF^ luciferase reporter (Fig. 4b lane 3 to 5), co-expression of CDK5RAP3 did not affect the E2F1-mediated *p14^ARF^* transactivation (Fig. 4b lane 6 to 8). Therefore, the mechanism by which CDK5RAP3 downregulates p14^ARF^ promoter transcription is most likely independent of E2F1.

**Figure 5:**
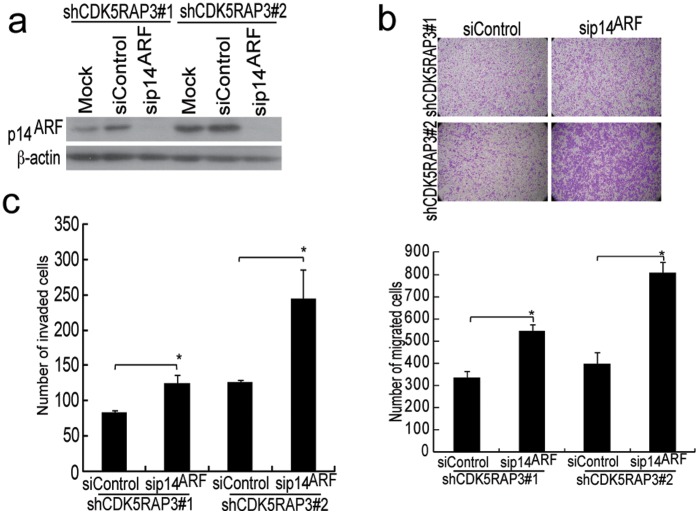
Knockdown of p14^ARF^ reversed the suppression of cell migration and invasiveness in CDK5RAP3 knockdown HCC cells. (**a**) The CDK5RAP3 stable knockdown SMMC-7721 cells were transfected with p14^ARF^ or control siRNA. *Top,* Western blotting showing p14^ARF^ knockdown in cells; *bottom,* The bar chart showed the quantitation of migrated cells in three independent experiment (*, *P* = 0.005, Student’s *t*-test). Representative photomicrographs were shown. (**b**) Similar to **a**), but invasion assay were performed. The bar chart showed the quantitation of the invaded cells in three independent experiment (*, *P* = 0.05, Student’s *t*-test).

### Knockdown of p14^ARF^ Reversed the Suppression of Cell Migration and Invasiveness in CDK5RAP3 Knockdown cells

Since evidence has demonstrated that mouse homolog of p14^ARF^, p19^ARF^ inhibited HCC cell invasion [16], we speculate that the upregulation of p14^ARF^ in CDK5RAP3 stable knockdown HCC clones may cause the decrease in migration of these stable clones [5]. To elucidate whether p14^ARF^ plays a role in CDK5RAP3-mediated regulation of invasiveness in HCC cells, we examine the effect of silencing p14^ARF^ in CDK5RAP3 stable knockdown clones motility. The specific knockdown of p14^ARF^ by two independent p14^ARF^ siRNA was confirmed by Western blotting (Fig. 5a). Then we asked if the effect of knockdown of CDK5RAP3 on cell motility and invasiveness could be restored by knockdown of p14^ARF^. As shown in Fig. 5b, knockdown of p14^ARF^ in CDK5RAP3 stable knockdown SMMC-7721 cells significantly increased the number of migrated cells as compared to the control siRNA, suggesting that loss of p14^ARF^ reversed the suppression of cell migration. Consistently, the invasiveness of CDK5RAP3 stable knockdown SMMC-7721 cells was also significantly restored in p14^ARF^ siRNA knockdown cells (Fig. 5b). Thus these results demonstrated that overexpression of CDK5RAP3 can promote HCC cell metastasis through downregulation of *p14^ARF^*.

## Discussion

CDK5RAP3 has 2 putative LXXLL motifs, which are the signature motifs for transcriptional co-regulators, mediating the binding on nuclear receptors. Previous study has shown that CDK5RAP3 can associate with a nuclear co-activator, cAMP response element-binding protein (CREB)-binding protein (CBP), suggesting that CDK5RAP3 may also function as a transcriptional co-activator/repressor. Recently, *CDK5RAP3* is shown to promote HCC metastasis by activating PAK4 kinase activity [5]. In this study, we provide evidence for a novel mechanism by which CDK5RAP3 promotes HCC metastasis by downregulating tumor suppressor p14^ARF^ transcriptionally. Our data demonstrated that loss of CDK5RAP3 drastically enhanced the expression of *p14^ARF^* (Fig. 1a and 2a) at both protein and mRNA levels in HCC cells. This notion is supported by three line of evidence. First, we observed that upregulation of *p14^ARF^* transcripts was observed in CDK5RAP3 stable knockdown HCC clones, whereas downregulation of *p14^ARF^* transcripts was observed in CDK5RAP3 stable overexpressing HCC clones. Second, using *p14^ARF^* promoter luciferase reporter assay, we showed that forced expression of CDK5RAP3 repressed the *p14^ARF^* promoter transcriptional activity in a dose-dependent manner (Fig. 2c). Third, using ChIP analysis, CDK5RAP3 was found to bind directly to *p14^ARF^* promoter region (Fig. 2e), indicating that CDK5RAP3 may directly regulate the p14^ARF^ promoter activity. Indeed, CDK5RAP3 has recently been reported to repress cyclin D1 transcription [17] and NF-κB transcriptional activity [18], further supporting the role of CDK5RAP3 as a transcriptional co-regulator. Taken together, these data indicated that CDK5RAP3 is a putative negative transcriptional regulator of *p14^ARF^*. As for how CDK5RAP3 can repress the p14^ARF^ promoter activity, we find that the overall integrity of the protein may be important. It is because all the truncation of CDK5RAP3 resulted in a completely lost of repressive activity of CDK5RAP3, apart from 1–362 mutant, which retains a moderate repressive activity (Fig. 3b). Interestingly, our data also suggested that the nuclear localization of CDK5RAP3 is important for its repressor activity (Fig. 3d) and this supports our hypothesis that CDK5RAP3 regulates the transcription of p14^ARF^ via direct binding to p14^ARF^ promoter, as revealed by our ChIP analysis (Fig. 2e).

p14^ARF^ is one of the upstream activators for p53 as it can abrogate the MDM2-mediated degradation of p53 by inhibiting MDM2 [14], [15]. However, in CDK5RAP3 stable knockdown HCC cells, we found that p53 and phospho-p53 (Ser15) levels remained unchanged (Fig. 1a). Furthermore, overexpression of CDK5RAP3 in HCC cells did not seem to have a significant impact on p53 transactivation activity (Fig. 4a). Thus these results indicate that CDK5RAP3 did not play a significant role in regulating p53 via p14^ARF^ in HCC cells. Actually, this observation is in consistent with our previous result showing that the transforming ability of CDK5RAP3 on HCC cells were p53-independent as CDK5RAP3 knockdown reduced proliferation rate and colonies formed in p53-defective PLC/PRF/5 HCC cell line [5].

The mechanism by which overexpression of CDK5RAP3 enhances HCC metastasis is not completely understood. Here, we propose that via the downregulation of p14^ARF^, CDK5RAP3 can enhance the invasiveness of HCC cells. To this end, we used siRNA to specifically knock down p14^ARF^ in SMMC-7721 cells with CDK5RAP3 stable knockdown and showed that the loss of p14^ARF^ significantly promoted the motility and invasiveness of HCC cells (Fig. 5). Previous studies have shown that mouse homolog of p14^ARF^, p19^ARF^ can inhibit the invasion of HCC cells by binding to C-terminal binding protein (CtBP) [16]. Interestingly, while CtBP binds to a.a. residues 42 to 54 of p14^ARF^ [16], CDK5RAP3 binds to a.a. residues 1 to 64 of p14^ARF^ [4], suggesting that the p14^ARF^ binding region of CtBP and CDK5RAP3 may indeed overlap. Thus it is conceivable that CDK5RAP3 may sequester the binding of p14^ARF^ to CtBP and release the free CtBPs to promote HCC cell invasion. Further experiment is currently undergoing to test this hypothesis.

Collectively, the underlying mechanism for the transcriptional regulation of *p14^ARF^* by CDK5RAP3 still not completely clear, and whether CDK5RAP3 regulates generally on *INK4a/p14^ARF^/INK4b* locus or specifically on *p14^ARF^* requires further investigation. As CDK5RAP3 has been reported to interact with CBP [19], a co-activator for CREB, it remains to be determined whether CDK5RAP3 regulates *p14^ARF^* promoter by modulating CBP/CREB activity. In HCC, silencing of the *p14^ARF^* promoter through hypermethylation is frequently observed [20]. However, our data suggest that *p14^ARF^* transcription can also be repressed by upregulation of CDK5RAP3. In addition, our data provide a novel insight by which overexpression of CDK5RAP3 can enhance HCC metastasis via negatively regulating *p14^ARF^*. As a result, inhibition of CDK5RAP3 can potentially be used to restore the expression of this important tumor suppressor expression, providing new molecular targets for the therapeutic intervention in HCC.

## References

[1] Ching YP, Qi Z, Wang JH (2000) Cloning of three novel neuronal Cdk5 activator binding proteins. Gene 242: 285–294. 10721722 10.1016/s0378-1119(99)00499-0

[2] Jiang H, Luo S, Li H (2005) Cdk5 activator-binding protein C53 regulates apoptosis induced by genotoxic stress via modulating the G2/M DNA damage checkpoint. J Biol Chem 280: 20651–20659. 15790566 10.1074/jbc.M413431200

[3] Gusarova GA, Wang IC, Major ML, Kalinichenko VV, Ackerson T, et al (2007) A cell-penetrating ARF peptide inhibitor of FoxM1 in mouse hepatocellular carcinoma treatment. J Clin Invest 117: 99–111. 17173139 10.1172/JCI27527 PMC1697798

[4] Wang J, He X, Luo Y, Yarbrough WG (2006) A novel ARF-binding protein (LZAP) alters ARF regulation of HDM2. Biochem J 393: 489–501. 16173922 10.1042/BJ20050960 PMC1360699

[5] Mak GW, Chan MM, Leong VY, Lee JM, Yau TO, et al (2011) Overexpression of a novel activator of PAK4, the CDK5 kinase associated protein CDK5RAP3, promotes hepatocellular carcinoma metastasis. Cancer Research 71: 2949–2958. 21385901 10.1158/0008-5472.CAN-10-4046

[6] Agesen TH, Florenes VA, Molenaar WM, Lind GE, Berner JM, et al (2005) Expression patterns of cell cycle components in sporadic and neurofibromatosis type 1-related malignant peripheral nerve sheath tumors. J Neuropathol Exp Neurol 64: 74–81. 15715087 10.1093/jnen/64.1.74

[7] Komori H, Enomoto M, Nakamura M, Iwanaga R, Ohtani K (2005) Distinct E2F-mediated transcriptional program regulates p14^ARF^ gene expression. Embo J 24: 3724–3736. 16211008 10.1038/sj.emboj.7600836 PMC1276720

[8] Zhou D, Jiang S, Shen Z, Gu J (1996) Effect of all-trans-retinoic acid and phorbol 12-myristate 13-acetate on the activity of human hepatocellular carcinoma cell-surface beta-1,4-galactosyltransferase. Biochem J 320 (Pt 2): 623–625. 10.1042/bj3200623 PMC1217975 8973576

[9] Ching YP, Leong VY, Lee MF, Xu HT, Jin DY, et al (2007) P21-activated protein kinase is overexpressed in hepatocellular carcinoma and enhances cancer metastasis involving c-Jun NH2-terminal kinase activation and paxillin phosphorylation. Cancer Res 67: 3601–3608. 17440071 10.1158/0008-5472.CAN-06-3994

[10] Leung TH, Ching YP, Yam JW, Wong CM, Yau TO, et al (2005) Deleted in liver cancer 2 (DLC2) suppresses cell transformation by means of inhibition of RhoA activity. Proc Natl Acad Sci U S A 102: 15207–15212. 16217026 10.1073/pnas.0504501102 PMC1250229

[11] Wong CC, Wong CM, Tung EK, Man K, Ng IO (2009) Rho-kinase 2 is frequently overexpressed in hepatocellular carcinoma and involved in tumor invasion. Hepatology 49: 1583–1594. 19205033 10.1002/hep.22836

[12] Kuo ML, den Besten W, Bertwistle D, Roussel MF, Sherr CJ (2004) N-terminal polyubiquitination and degradation of the Arf tumor suppressor. Genes Dev 18: 1862–1874. 15289458 10.1101/gad.1213904 PMC517406

[13] Robertson K, Jones P (1998) The Human ARF Cell Cycle Regulatory Gene Promoter Is a CpG Island Which Can Be Silenced by DNA Methylation and Down-Regulated by Wild-Type p53. Molecular and Cellular Biology 18: 6457–6473. 9774662 10.1128/mcb.18.11.6457 PMC109232

[14] Moll UM, Petrenko O (2003) The MDM2-p53 Interaction. Molecular Cancer Research 1: 1001–1008. 14707283

[15] Weber JD, Taylor LJ, Roussel MF, Sherr CJ, Bar Sagi D (1999) Nucleolar Arf sequesters Mdm2 and activates p53. Nat Cell Biol 1: 20–26. 10559859 10.1038/8991

[16] Chen YW, Paliwal S, Draheim K, Grossman SR, Lewis BC (2008) p19^Arf^ inhibits the invasion of hepatocellular carcinoma cells by binding to C-terminal binding protein. Cancer Res 68: 476–482. 18199542 10.1158/0008-5472.CAN-07-1960 PMC2376045

[17] Shiwaku H, Yoshimura N, Tamura T, Sone M, Ogishima S, et al (2010) Suppression of the novel ER protein Maxer by mutant ataxin-1 in Bergman glia contributes to non-cell-autonomous toxicity. Embo J 29: 2446–2460. 20531390 10.1038/emboj.2010.116 PMC2910266

[18] Wang J, An H, Mayo MW, Baldwin AS, Yarbrough WG (2007) LZAP, a putative tumor suppressor, selectively inhibits NF-kappaB. Cancer Cell 12: 239–251. 17785205 10.1016/j.ccr.2007.07.002

[19] Yin X, Warner DR, Roberts EA, Pisano MM, Greene RM (2005) Novel interaction between nuclear co-activator CBP and the CDK5 activator binding protein - C53. Int J Mol Med 16: 251–256. 16012757

[20] Anzola M, Cuevas N, Lopez-Martinez M, Saiz A, Burgos JJ, et al (2004) p14^ARF^ gene alterations in human hepatocellular carcinoma. Eur J Gastroenterol Hepatol 16: 19–26. 15095848 10.1097/00042737-200401000-00004

